# Smart Fluids As Autophagy-Activating Photoprotectors: In Vitro Analysis of Dead Sea Water and Magnetized Saline Water Against Ultraviolet B (UVB)-Induced Photodamage in Human Keratinocytes

**DOI:** 10.7759/cureus.82224

**Published:** 2025-04-14

**Authors:** Eugenia V Di Brizzi, Simone Lista, Celia García-Chico, Kayvan Khoramipour, Alejandro Santos-Lozano, Piercarlo Minoretti

**Affiliations:** 1 Dermatology, University of Campania "Luigi Vanvitelli", Naples, ITA; 2 Health Sciences, Miguel de Cervantes European University, Valladolid, ESP; 3 Occupational Health, Studio Minoretti, Oggiono, ITA

**Keywords:** autophagy, cyclobutane pyrimidine dimers, dead sea water, inflammation, keratinocytes, magnetized water, ultraviolet radiation

## Abstract

Background

Autophagy induction has been shown to mitigate both ultraviolet B (UVB)-induced DNA damage and inflammation. Smart fluids, including Dead Sea water (DSW) and saline magnetized water (MW), have recently been suggested to promote autophagy activation. This in vitro study was designed to investigate the ability of DSW and saline MW to inhibit the formation of UVB-induced cyclobutane pyrimidine dimers (CPDs) and the expression of the NOD-like receptor protein 3 (NLRP3) inflammasome in UVB-irradiated HaCaT cells, a well-established, spontaneously immortalized human keratinocyte cell line.

Methods

To explore whether autophagy mediated the photoprotection induced by smart fluids, we measured two established autophagy markers (beclin-1 and LC3B) in HaCaT cell lysates and examined how wortmannin, an autophagy inhibitor, modulated the smart fluids’ effects on post-irradiation CPDs and NLRP3 inflammasome levels.

Results

Compared to unirradiated control cells not exposed to any fluid (set at 1 a.u.), pretreatment with DSW (15.7 ± 1.9 a.u.) and saline MW (11.3 ± 1.6 a.u.) markedly reduced CPD formation in UVB-irradiated cells compared to two control fluids (saline non-MW: 20.9 ± 0.8 a.u.; distilled water: 21.4 ± 0.6 a.u.) (all p < 0.001). Notably, among the two smart fluids, saline MW significantly outperformed DSW in terms of DNA protection (p < 0.001). Conversely, DSW and saline MW demonstrated no statistically significant difference in NLRP3 inflammasome inhibition (p = 0.56). Both smart fluids effectively attenuated the UVB-induced decrease in beclin-1 and LC3B (all *p* < 0.001), although the observed effects were significantly more pronounced for saline MW (both p < 0.05). Notably, wortmannin either partially (CPDs) or completely (NLRP3 inflammasome) abrogated the photoprotective effects of both DSW and saline MW, suggesting that the observed chemopreventive properties were mainly attributable to their action as autophagy activators.

Conclusions

Our findings support the potential application of DSW and saline MW as sustainable active ingredients in topical skin products aimed at preventing UVB-induced non-melanoma skin cancers and cutaneous inflammaging.

## Introduction

The skin is the largest organ in the human body and acts as a protective barrier against various hazardous environmental factors, including solar ultraviolet radiation (UVR) [1]. Of the UVR reaching the Earth’s surface, 95% is UVA (320-400 nm) and 5% is UVB (290-320 nm) [2]. While UVA radiation can penetrate both the epidermis and dermis, it is not directly absorbed by DNA and can only induce oxidative DNA damage through the indirect production of reactive oxygen species [3]. Conversely, UVB is primarily absorbed by epidermal keratinocytes and can induce direct chemical modifications in DNA via the formation of specific photoproducts, the most common being cyclobutane pyrimidine dimers (CPDs) [4]. Notably, CPDs are the main molecular precursors of UVR-signature DNA mutations (i.e., C>T and CC>TT), which represent 25% and 75% of the total mutational events identified in malignant melanoma and non-melanoma skin cancers (NMSCs), respectively [5]. Besides inducing direct DNA damage through CPD formation, UVB can also promote inflammation through the activation of the NOD-like receptor protein 3 (NLRP3) inflammasome [6], a multiprotein complex that triggers the release of numerous inflammatory mediators [7]. Consequently, novel strategies that can be used in combination with traditional sunscreens to curb UVB-induced photodamage are of major relevance for preventing both NMSCs and skin inflammaging [8]. Autophagy, a cellular recycling system that mediates the turnover of aggregated or damaged proteins [9], is a highly conserved defense mechanism that keratinocytes employ for protection against UVB-induced photodamage [10, 11]. While UVB exposure inhibits autophagy in keratinocytes, autophagy activation prevents UVB-induced cell death [10-12]. Importantly, autophagy induction has been previously shown to mitigate both UVB-induced DNA damage and inflammation [13, 14]. Therefore, topical strategies that activate autophagy in keratinocytes hold significant promise for enhancing current photoprotection strategies [4, 10, 12].

Dead Sea water (DSW), a natural smart fluid with a unique mineral composition rich in magnesium, calcium, sodium, potassium, zinc, sulphides, strontium, and bromides, has been extensively investigated in dermatology due to its barrier repair, anti-inflammatory, and anti-photoaging properties [15, 16]. Recently, Yan X et al. [17] used human dermal cells and reconstructed skin models to demonstrate that DSW treatment can activate, among other pathways, DNA repair and autophagy-related molecular mechanisms. Apart from DSW, autophagy-activating properties have also been reported for another smart fluid, namely magnetized water (MW) [18, 19], i.e., water subjected to magnetic field (MF) treatment [20]. Notably, MF exposure can induce significant changes in the properties of water, despite its diamagnetic nature [21]. These modifications include reduced surface tension, increased pH levels and shear viscosity, and enhanced electrical conductivity [22, 23]. While MW has already found established applications in agriculture [24] and the construction industry [25], its medical potential remains largely unexplored. However, emerging evidence suggests that topically applied saline MW may improve skin biophysical parameters [18] and accelerate wound healing [26] by activating autophagy topically.

Considering the importance of autophagy in inhibiting UVB-induced cellular damage, we designed an in vitro study to investigate the photoprotective potential of two smart fluids, DSW and saline MW, through two main objectives. First, we evaluated their chemopreventive properties by comparing their capacity to suppress UVB-induced CPD formation and NLRP3 inflammasome expression in irradiated HaCaT cells, a well-established, spontaneously immortalized human keratinocyte cell line. Second, we investigated whether autophagy mediated the observed photoprotective effects. Specifically, we analyzed two autophagy markers (beclin-1 and LC3B) in cell lysates and assessed how wortmannin, a known autophagy inhibitor, modulated the effects of smart fluids on post-irradiation CPD and NLRP3 inflammasome levels [19, 27].

## Materials and methods

Materials

Two smart fluids, DSW and saline MW, and two control fluids, saline non-MW and distilled water (DW), were utilized in all experiments. DSW (BIO-ROM s.r.o., Bratislava, Slovakia) was procured from an online retailer. Saline MW was sourced from Aquavis srl (Brescia, Italy). The production process involved the preparation of a patented saline water solution, containing 0.9% NaCl, 0.011% KCl₂, 0.009% CaCl₂, 0.007% MgCl₂, 0.007% ZnCl₂, and 0.007% AlCl₂, which was subsequently exposed to a MF of fixed strength (3000 Gauss) for two hours. The resulting saline MW was stored at room temperature and used within three months of preparation. For control purposes, a batch of saline water with identical salt concentrations but not exposed to a MF was used. Molecular biology-grade DW (Sigma, St. Louis, MO, USA) served as an additional control fluid.

Experimental protocol

**Figure 1:**
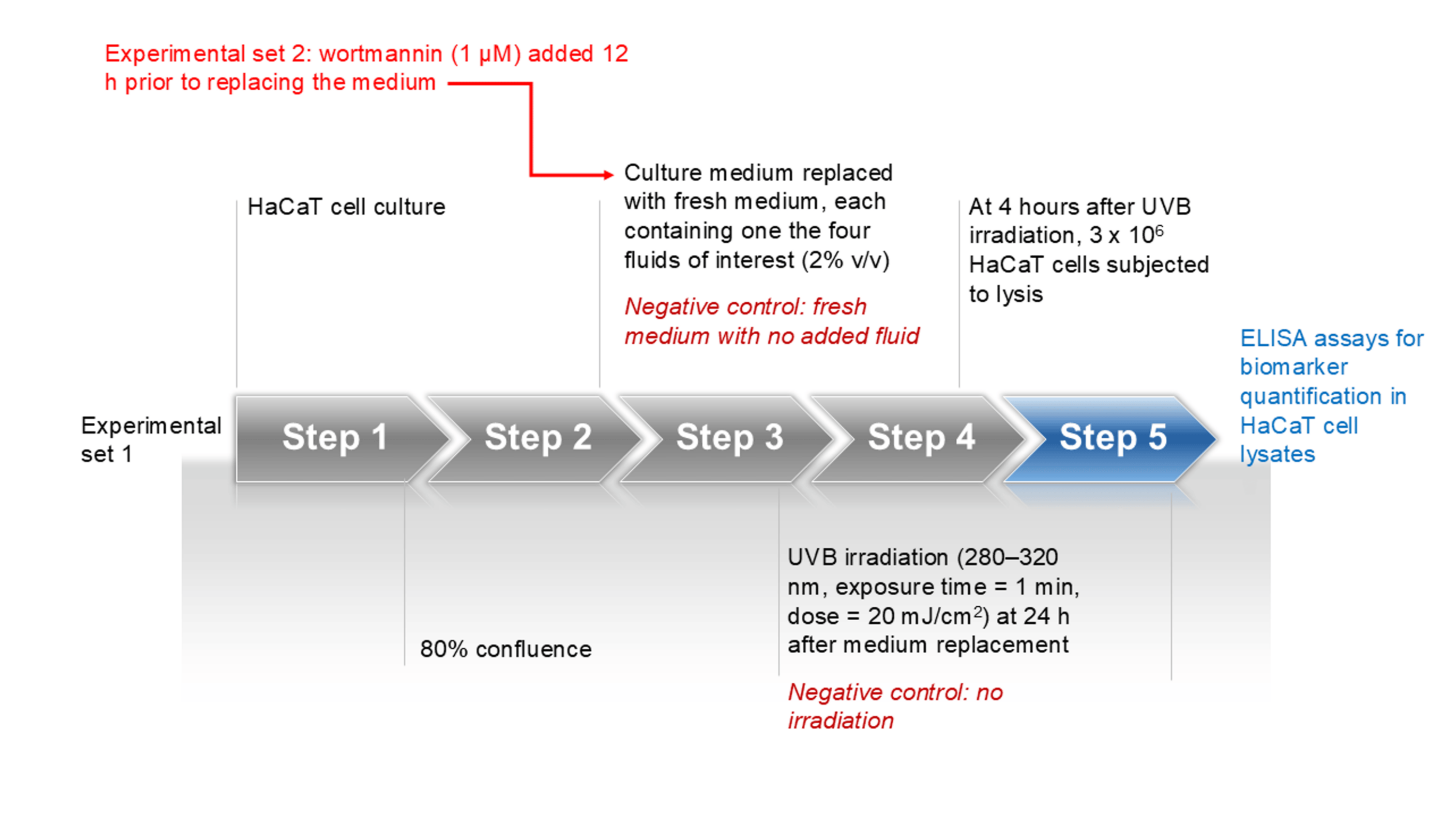
Schematic representation of the experimental protocol.

HaCaT keratinocytes were obtained from CLS Cell Lines Service GmbH (Eppelheim, Germany) and cultured in Dulbecco's Modified Eagle’s Medium (DMEM; Sigma), supplemented with 10% fetal bovine serum and 1% penicillin/streptomycin under standard cell culture conditions (37 °C, 5% CO₂ in a humidified incubator). The cells were grown until they reached 80% confluence. Two sets of experiments were designed. In the first round, 24 hours prior to UVB irradiation, the medium was replaced with fresh medium containing one of the following fluids: (1) DSW at a concentration of 2% v/v; (2) Saline MW at 2% v/v; (3) Saline non-MW at 2% v/v; and (4) DW at 2% v/v. The cells were subsequently exposed to UVB radiation at a dose of 20 mJ/cm² for 1 minute using a Spectrolinker XL-1500 UV crosslinker (Spectronics, Westbury, NY, USA), which emits mainly in the UVB range (280-320 nm; peak: 312 nm). Cells that received no fluid pretreatment and were not exposed to UVB irradiation served as negative controls. In the second set of experiments, to investigate whether autophagy activation induced in HaCaT cells by smart fluids could play a role in photoprotection, all procedures were repeated under the same conditions but with the addition of the autophagy inhibitor wortmannin (1 μM; Sigma), 12 hours prior to replacing the culture medium with fresh medium (Figure 1).

Biomarker quantification in HaCaT cell lysates

At four hours after UVB irradiation, HaCaT culture samples, containing approximately 3 × 10⁶ cells, underwent lysis in an appropriate buffer (200 μL), followed by a 10-minute centrifugation at 13,000 × g. NLRP3, beclin-1, and LC3B protein concentrations in HaCaT cell lysates were measured using commercially available ELISA kits (MyBioSource Inc., San Diego, CA, USA) according to the manufacturer's protocol. For the quantification of CPDs, samples were subjected to enzymatic digestion with proteinase K in a buffer solution composed of 100 mmol/L Tris-HCl (pH 7.4), 150 mmol/L NaCl, and 10 mmol/L EDTA (pH 8.0). The digestion process lasted for 12 hours at 60 °C, after which the enzyme was heat-inactivated at 95 °C for 10 minutes. Subsequently, DNA was isolated using a commercially available extraction kit (Qiagen, Hilden, Germany). Finally, CPDs were quantified with a specific ELISA kit (OxiSelect Cellular UV-Induced DNA Damage ELISA Kit; Cell Biolabs, San Diego, CA, USA) [4].

Data analysis

All biomarker measurements are expressed in arbitrary units (a.u.), with unirradiated control cells not exposed to any fluid serving as the reference, conventionally set at 1. Continuous data are presented as the mean ± SD of at least three independent experiments, each conducted in duplicate. To compare the different experimental conditions, an unpaired one-way ANOVA was performed, followed by Tukey's post hoc test to identify significant differences between groups. Analyses were carried out using SPSS, version 20.0 (IBM, Armonk, NY, USA), and two-tailed p values < 0.05 were considered statistically significant.

## Results

Smart fluids inhibit UVB-induced DNA damage and inflammation in HaCaT cells

Following UVB irradiation, HaCaT cells exposed to DW exhibited significantly elevated levels of CPDs and NLRP3 inflammasome (21.4-fold and 2.64-fold increases, respectively) compared to unirradiated control cells not exposed to any fluid (Table 1).

**Table 1:** Cyclobutane pyrimidine dimers and NLRP3 inflammasome levels in HaCaT cells exposed to different experimental conditions. UVB: Ultraviolet B; CPDs: Cyclobutane pyrimidine dimers; NLRP3: NOD-like receptor protein 3. Data are presented as means ± standard deviation of at least three independent experiments. The negative control cells were conventionally set at 1. One-way ANOVA showed significant differences between groups for CPDs (F(4,10) = 42.68, p < 0.001) and NLRP3 inflammasome (F(4,10) = 15.23, p < 0.001). * p < 0.001 versus distilled water and saline non-magnetized water (post hoc Tukey’s test); † p < 0.001 versus Dead Sea water (post hoc Tukey’s test).

	Negative control	Distilled water	Saline non-magnetized water	Dead Sea water	Saline magnetized water
UVB irradiation	No	Yes	Yes	Yes	Yes
CPDs, a.u.	1	21.4 ± 0.6	20.9 ± 0.8	15.7 ± 1.9*	11.3 ± 1.6*,†
NLRP3 inflammasome, a.u.	1	2.64 ± 0.3	2.34 ± 0.8	1.67 ± 0.5*	1.72 ± 0.5*,†

Cells treated with saline non-MW showed comparable increases for both biomarkers (20.9- and 2.34-fold for CPDs and NLRP3 inflammasome, respectively), without significant differences compared to DW. In contrast, the two smart fluids demonstrated significant protective effects against UVB-induced DNA damage and inflammation. Specifically, DSW (15.7 ± 1.9 a.u.) and saline MW (11.3 ± 1.6 a.u.) markedly reduced CPD formation in UVB-irradiated cells compared to the two control fluids (saline non-MW: 20.9 ± 0.8 a.u.; DW: 21.4 ± 0.6 a.u.) (all p < 0.001). Notably, among the two smart fluids, saline MW demonstrated superior efficacy in reducing post-UVB irradiation CPD formation compared to DSW (p < 0.001). However, regarding post-UVB irradiation NLRP3 inflammasome levels in HaCaT cells, DSW (1.67 ± 0.5 a.u.) and saline MW (1.72 ± 0.5 a.u.) showed similar anti-inflammatory effects (p = 0.56), both significantly outperforming the two control fluids (saline non-MW: 2.34 ± 0.8 a.u.; DW: 2.64 ± 0.3 a.u.; all p < 0.001).

Smart fluids attenuate UVB-induced autophagy inhibition in cultured keratinocytes

Subsequently, we investigated the levels of two autophagy biomarkers, beclin-1 and LC3B, in HaCaT cell lysates under different experimental conditions (Table 2).

**Table 2:** Levels of autophagy biomarkers in HaCaT cells exposed to different experimental conditions. UVB: Ultraviolet B. Data are presented as means ± SD of at least three independent experiments. The negative control cells were conventionally set at 1. One-way ANOVA showed significant differences between groups for beclin-1 (F(4,10) = 18.72, p < 0.001) and LC3B (F(4,10) = 16.35, p < 0.001). * p < 0.001 versus distilled water and saline non-magnetized water (post hoc Tukey’s test); † p < 0.001 versus Dead Sea water (post hoc Tukey’s test).

	Negative control	Distilled water	Saline non-magnetized water	Dead Sea water	Saline magnetized water
UVB irradiation	No	Yes	Yes	Yes	Yes
Beclin-1, a.u.	1	0.60 ± 0.1	0.62 ± 0.1	0.77 ± 0.1*	0.94 ± 0.2*,†
LC3B, a.u.	1	0.61 ± 0.2	0.59 ± 0.2	0.80 ± 0.1*	0.97 ± 0.2*,†

As expected, following UVB irradiation, cells exposed to DW showed significant reductions in both beclin-1 (0.60 ± 0.1 a.u.) and LC3B (0.61 ± 0.2 a.u.) levels compared to non-irradiated negative controls. Similar decreases were observed in cells treated with saline non-MW (beclin-1: 0.62 ± 0.1 a.u.; LC3B: 0.59 ± 0.2 a.u.), with no significant differences between the two control fluids (p = 0.84). However, pretreatment with the two smart fluids significantly attenuated UVB-induced autophagy inhibition. Specifically, DSW maintained higher levels of both beclin-1 (0.77 ± 0.1 a.u.) and LC3B (0.80 ± 0.1 a.u.) compared to both control fluids (all p < 0.001). Notably, saline MW demonstrated significantly greater autophagy-preserving effects, maintaining near-normal levels of both beclin-1 (0.94 ± 0.2 a.u.) and LC3B (0.97 ± 0.2 a.u.), which were significantly higher than those observed with DSW (both p < 0.05).

Pharmacological inhibition of autophagy markedly impairs the photoprotective effects of smart fluids in cultured keratinocytes

As expected, pretreatment of HaCaT cells with the autophagy inhibitor wortmannin significantly suppressed the expression of beclin-1 and LC3B across all experimental conditions (Table 3).

**Table 3:** Levels of autophagy biomarkers, cyclobutane pyrimidine dimers, and NLRP3 inflammasome in HaCaT cells pretreated with wortmannin and exposed to different experimental conditions. UVB: Ultraviolet B; CPDs: Cyclobutane pyrimidine dimers; NLRP3: NOD-like receptor protein 3. Data are presented as means ± standard deviation of at least three independent experiments. The negative control cells were conventionally set at 1. One-way ANOVA showed significant differences between groups for beclin-1 (F(4,10) = 45.21, p < 0.001), LC3B (F(4,10) = 42.83, p < 0.001), CPDs (F(4,10) = 12.56, p < 0.01), and NLRP3 inflammasome (F(4,10) = 8.74, p < 0.01). * p < 0.05 versus distilled water and saline non-magnetized water (post hoc Tukey’s test).

	Negative control	Distilled water	Saline non-magnetized water	Dead sea water	Saline magnetized water
UVB irradiation	No	Yes	Yes	Yes	Yes
Beclin-1, a.u.	1	0.23 ± 0.1	0.22 ± 0.1	0.23 ± 0.1	0.25 ± 0.1
LC3B, a.u.	1	0.21 ± 0.1	0.25 ± 0.1	0.26 ± 0.1	0.27 ± 0.1
CPDs, a.u.	1	36.7 ± 3.4	37.1 ± 3.9	31.9 ± 4.1*	30.2 ± 3.9*
NLRP3 inflammasome, a.u.	1	3.15 ± 0.8	3.32 ± 0.9	3.09 ± 1.0	3.05 ± 0.9

In addition, wortmannin significantly exacerbated UVB-induced CPD formation and increased NLRP3 inflammasome levels in HaCaT cells compared to experiments conducted without its addition (Table 1, all p < 0.001). Notably, wortmannin substantially impaired the protective effects of both DSW and saline MW against UVB-induced CPD formation, although both smart fluids retained a modest DNA-protective effect compared to the two control fluids (both p < 0.05). However, the protective effect of both DSW and saline MW against UVB-induced inflammation in HaCaT cells, as reflected by NLRP3 inflammasome levels in cell lysates, was completely abrogated by wortmannin pretreatment.

## Discussion

In addition to traditional physical and chemical sunscreens, there is an ongoing search for novel, sustainable strategies to prevent UVB-induced DNA damage and skin inflammaging, given the rising global incidence of NMSCs [28]. In this study, we assessed the photoprotective effects of two environmentally friendly smart fluids, DSW and saline MW, on HaCaT keratinocytes experimentally exposed to UVB radiation. To this end, we specifically focused on CPDs [4] and the NLRP3 inflammasome [6] as biochemical markers of genotoxicity and inflammation, respectively. From a chemopreventive perspective, we found that pretreatment with both DSW and saline MW significantly reduced the formation of CPDs in DNA extracted from UVB-irradiated keratinocytes, with the protective effect being more pronounced for saline MW. Conversely, DSW and saline MW were similarly effective in inhibiting the NLRP3 inflammasome.

There is growing evidence that DSW may prevent UVB-induced skin inflammaging [29]. Using skin explants subjected to experimental irradiation, Cohen D et al. [30] demonstrated that co-culture with DSW inhibited UVB-induced apoptosis and reduced the release of proinflammatory interleukin (IL)-1β and IL-8. In a separate study, Soroka Y et al. [31] reported that minerals from DSW attenuated UVB-induced apoptotic damage in aged keratinocytes, while stimulating mitochondrial activity and cell proliferation. This preclinical evidence has led to the development of DSW-containing cosmetic preparations aimed at mitigating the visible signs of cutaneous inflammaging. Our current study provides substantial support for the notion that DSW can significantly inhibit the inflammatory response, as reflected by NLRP3 inflammasome concentrations, in UVB-irradiated HaCaT cells. However, to our knowledge, there have been no previous reports demonstrating that DSW can significantly reduce the burden of DNA photoproducts (namely, CPD levels) in irradiated keratinocytes. Autophagy plays a crucial role in maintaining genome stability and is activated in response to genotoxic insults [32]. We therefore hypothesized that DSW could exert its photoprotective effects, at least partially, by mitigating UVB-induced impairment of autophagic flux in keratinocytes. Separately, based on recent reports of saline MW activating autophagy when applied topically to human skin [18, 19], we compared both smart fluids not only for their photoprotective effects but also for their ability to counteract reductions in autophagy-related proteins in UVB-exposed HaCaT cells. Interestingly, our findings revealed that both smart fluids effectively attenuated the irradiation-induced decrease in beclin-1 and LC3B, although the observed effect was significantly more pronounced for saline MW. Considering that saline MW similarly outperformed DSW in reducing the CPDs burden following experimental irradiation, it is reasonable to hypothesize that the DNA-protective properties of the two smart fluids are unlikely the result of direct DNA repair. Instead, their effectiveness may be attributed to their capacity to maintain optimal function of the endogenous DNA repair machinery by sustaining a physiological autophagic flux [33]. Similar observations can be made regarding the attenuation of inflammation in keratinocytes exposed to UVB radiation following pretreatment with smart fluids. As a cytoplasmic degradative pathway, autophagy plays a critical role in mitigating excess inflammation by downregulating several inflammatory proteins and signaling pathways [34]. Consistently, wortmannin either partially (CPDs) or completely (NLRP3 inflammasome) abrogated the photoprotective effects of both DSW and saline MW, suggesting that the observed chemopreventive properties are mainly attributable to their action as autophagy activators. However, the mechanisms by which smart fluids may increase autophagic flux remain speculative. Hypersaline waters like DSW are known to induce ionic osmotic stress, against which autophagy is considered an adaptive mechanism [17]. Similarly, the alkaline stress elicited by saline MW is another potential pathway leading to autophagy activation [18, 19, 23]. Given that saline non-MW neither exhibited photoprotective effects nor activated autophagy, it is plausible that the increased shear viscosity, electrical conductivity, and pH levels induced by MF treatment in water [35] may play a role in activating autophagy-related molecular pathways.

Limitations

Despite the promising findings of our study, several limitations should be acknowledged. First, the in vitro design using HaCaT keratinocytes, while providing valuable mechanistic insights, may not fully replicate the complex physiological environment of human skin. The three-dimensional structure of skin, with its multiple cell types and extracellular matrix, could influence the photoprotective effects of smart fluids in ways not captured by our monolayer cell culture model. Second, our experiments focused on a single UVB dose (20 mJ/cm²), which may not represent the variable solar radiation exposures encountered in real-life settings. Future studies should examine the photoprotective effects of smart fluids across different UVB doses and potentially include UVA radiation to better simulate natural sunlight exposure. Finally, while we demonstrated that autophagy plays a significant role in the photoprotective effects of DSW and saline MW, we did not fully elucidate the precise molecular underpinnings by which saline MW and DSW may promote an increased autophagic flux. Additional studies employing molecular biology techniques such as gene silencing would provide more detailed insights into the signaling pathways involved.

## Conclusions

The present study indicates that smart fluids may exert significant photoprotective effects against UVB-induced DNA damage and inflammaging by promoting autophagy in HaCaT keratinocytes. Our findings support the potential application of DSW and saline MW as active ingredients in topical skin products aimed at preventing UVB-induced NMSCs and inflammaging. However, given the in vitro design of this study, further in vivo research is necessary to confirm the clinical relevance of these findings and to explore the potential value of smart fluids in the chemoprevention of NMSCs in at-risk populations.
